# The role of artificial intelligence in aortic valve stenosis: a bibliometric analysis

**DOI:** 10.3389/fcvm.2025.1521464

**Published:** 2025-02-12

**Authors:** Shanshan Chen, Changde Wu, Zhaojie Zhang, Lingjuan Liu, Yike Zhu, Dingji Hu, Chenhui Jin, Haoya Fu, Jing Wu, Songqiao Liu

**Affiliations:** ^1^ Department of Respiratory and Critical Care Medicine, Second Affiliated Hospital of Xuzhou Medical University, Xuzhou Mining Group General Hospital, Xuzhou, Jiangsu, China; ^2^ Jiangsu Provincial Key Laboratory of Critical Care Medicine, Department of Critical Care Medicine, Trauma Center, Zhongda Hospital, School of Medicine, Southeast University, Nanjing, Jiangsu, China; ^3^ Department of Critical Care Medicine, Trauma Center, Nanjing Lishui People’s Hospital, Zhongda Hospital Lishui Branch, Nanjing, Jiangsu, China; ^4^ The First People’s Hospital of Lianyungang, The Lianyungang Clinical College of Nanjing Medical University, The First Affiliated Hospital of Kangda College of Nanjing Medical University, The Affiliated Lianyungang Hospital of Xuzhou Medical University, Lianyungang, Jiangsu, China

**Keywords:** artificial intelligence, machine learning, aortic valve stenosis, bibliometrics, clinical decision support

## Abstract

**Purpose:**

To explore the expanding role of artificial intelligence (AI) in managing aortic valve stenosis (AVS) by bibliometric analysis to identify research trends, key contributors, and the impact of AI on enhancing diagnostic and therapeutic strategies for AVS.

**Methods:**

A comprehensive literature review was conducted using the Web of Science database, covering publications from January 1990 to March 2024. Articles were analyzed with bibliometric tools such as CiteSpace and VOSviewer to identify key research trends, core authors, institutions, and research hotspots in AI applications for AVS.

**Results:**

A total of 118 articles were analyzed, showing a significant increase in publications from 2014 onwards. The results highlight the growing impact of AI in AVS, particularly in cardiac imaging and predictive modeling. Core authors and institutions, primarily from the U.S. and Germany, are driving research in this field. Key research hotspots include machine learning applications in diagnostics and personalized treatment strategies.

**Conclusions:**

AI is playing a transformative role in the diagnosis and treatment of AVS, improving accuracy and personalizing therapeutic approaches. Despite the progress, challenges such as model transparency and data security remain. Future research should focus on overcoming these challenges while enhancing collaboration among international institutions to further advance AI applications in cardiovascular medicine.

## 1 Introduction

Aortic Valve Stenosis (AVS) is a significant cardiac disease, particularly affecting the elderly. As the population ages, addressing AVS through early diagnosis and treatment becomes increasingly critical (1). However, the complexity of AVS often complicates clinical decision-making (2, 3). Artificial Intelligence (AI), including Machine Learning (ML) and Deep Learning (DL), offers advanced capabilities in data processing and pattern recognition, which can significantly improve diagnosis and treatment accuracy (4). AI applications in AVS have shown great promise in areas like medical imaging and risk assessment (3, 5–6). Recent bibliometric analyses have explored various aspects of aortic valve disease and its management (7, 8). However, while these studies provide valuable insights into the broader field of aortic valve disease, they do not specifically address the applications of AI in AVS diagnosis and treatment. This study aims to fill this gap systematically explore AI's role in enhancing AVS diagnosis and treatment through bibliometric analysis, identifying key contributors and trends to guide future research in cardiovascular care.

## 2 Materials and methods

### 2.1 Literature search strategies

A comprehensive literature search was conducted using the Web of Science database for articles published from January 1990 to March 2024, aiming to capture pertinent research developments in the 21st century. The keyword strategy in Table 1 employed the following combination: (“aortic valve stenosis” OR “aortic stenosis”) AND (“artificial intelligence” OR “machine learning”).

**Table 1:** Summary of data source and selection.

Category	Specific Standard Requirements
Research database	Web of Science core collection
Citation indexes	Science Citation Index Expanded(SCI-EXPANDED) and Social Sciences Citation Index(SSCI)
Searching period	January 1990 to March 2024
Language	“English”
Searching keywords	(“aortic valve stenosis” OR “aortic stenosis”) AND (“artificial intelligence” OR “machine learning”)
Publication types	“Article” and “Review Article”
Data extraction	Export with full records and cited references in plain text format
Sample size	118

### 2.2 Inclusion/exclusion criteria

**Figure 1:**
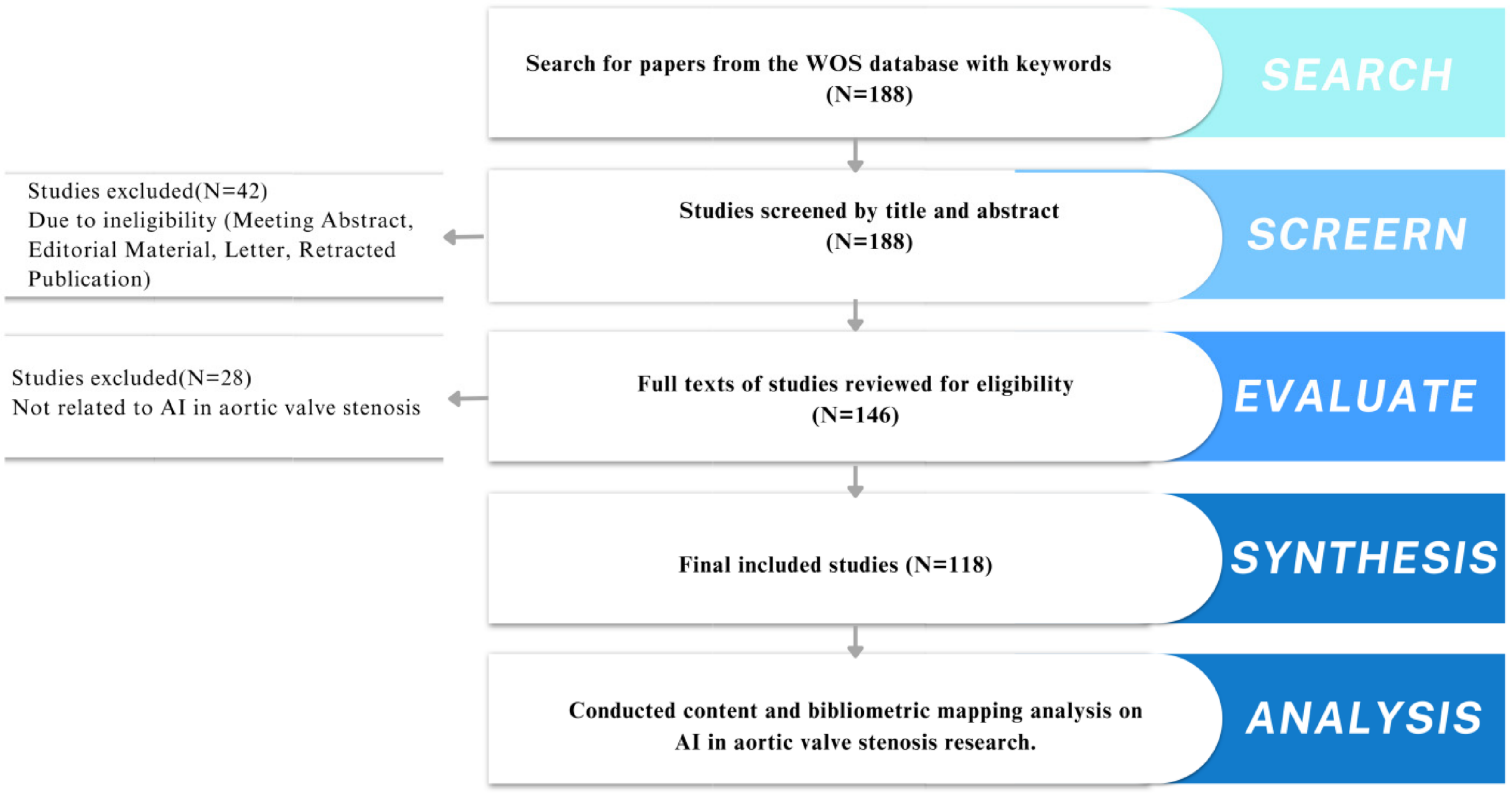
Flowchart of literature screening and research process.

Two independent researchers (Shanshan Chen and Lingjuan Liu) screened titles and abstracts of the retrieved articles (Figure 1). Inclusion criteria were: (1) studies discussing AI applications in aortic stenosis diagnosis or treatment, (2) original research or reviews, (3) full-text availability in English. Exclusion criteria included: (1) non-research articles like book chapters or conference abstracts, (2) duplicate studies. Any disagreements were resolved by a third reviewer (Songqiao Liu).

### 2.3 Tools and techniques for bibliometric analyses

CiteSpace and VOSviewer were used to analyze research trends, key authors, institutions, and geographic distributions. These tools provided visual representations of collaboration networks and emerging research hotspots. Data export and cleaning involved removing incomplete records, constructing networks of co-authorship and keyword co-occurrence, and identifying trends and collaboration patterns.

## 3 Results

### 3.1 Analysis of development trends

The study utilized 118 papers from 45 countries, 358 institutions, and 878 authors, published across 83 journals, and cited 3,929 references from 1,245 journals.

**Figure 2:**
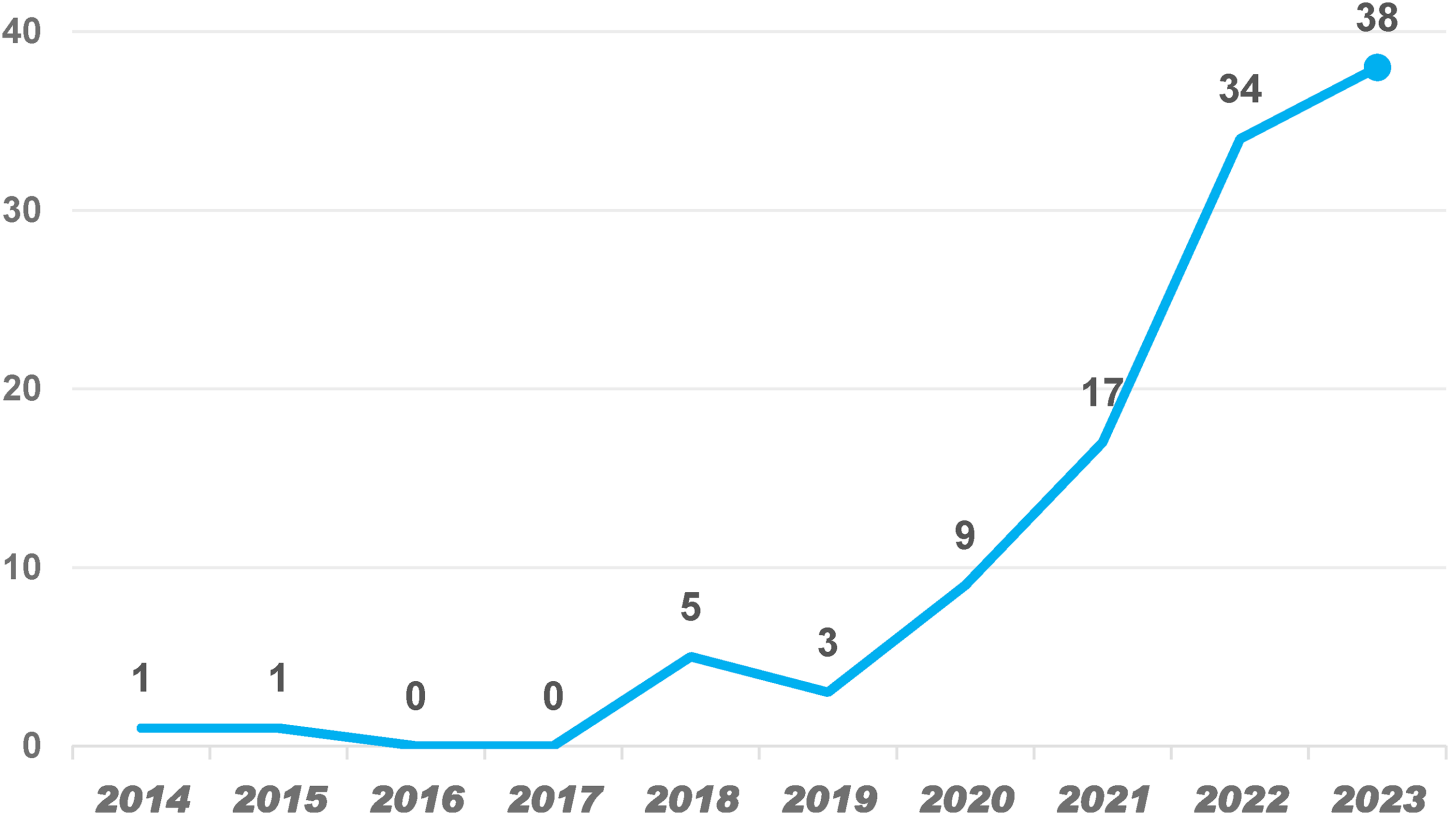
Trends in the growth of publications worldwide from 2014 to 2023.

From 118 papers across 45 countries and 358 institutions, the publication trend shows a steady increase in AI applications in AS research from 2014 to 2023 in Figure 2. A sharp rise occurred from 2020 to 2022, with a peak in 2023, indicating heightened interest driven by advancements in AI and increased clinical demand. The data suggests that AI research in AS will continue growing, particularly in diagnostics and personalized treatment. The record of only 10 publications in 2024 may be attributed to incomplete data collection; the publication volume is expected to increase as the year progresses.

Moreover, the analysis of development trends offers a macroscopic perspective, aiding in identifying the growth drivers of this research field and potential research gaps or future directions, such as the potential applications of AI technology in improving diagnostic accuracy, reducing misdiagnosis rates, and designing personalized treatment plans.

### 3.2 Analysis of authors and research institutions

#### 3.2.1 Identifying core authors

Using CiteSpace, we identified 12 core authors contributing 39 papers, which account for 33% of the total publication volume following Price's Law, indicating that the field has yet to form a stable group of authors. Average Citations per Item (ACI) serves as an indicator to measure the impact of scientific literature, commonly utilized to quantify the average number of citations received by a scholar, a journal, or an article, and Table 2 displays the highly productive authors in this field with more than three publications, ranked by the number of citations.

**Table 2:** Most important authors ranked by citations in AI applications in the aortic valve stenosis research field.

Rank	Author	Publications	Citations	ACI
1	Aranoff, Nicole D.	3	41	13.66
2	Green, Philip	3	41	13.66
3	Tavassolian, Negar	3	41	13.66
4	Dweck, Marc R.	3	38	12.66
5	Schoepf, U. Joseph	4	36	9
6	Emrich, Tilman	3	32	10.66
7	Batra, Puneet	3	25	8.33
8	Choi, Seung Hoan	3	25	8.33
9	Di Achille, Paolo	3	25	8.33
10	Nauffal, Victor	3	25	8.33
11	Renker, Matthias	3	17	5.66
12	Varga-Szemes, Akos	3	3	1

**Figure 3:**
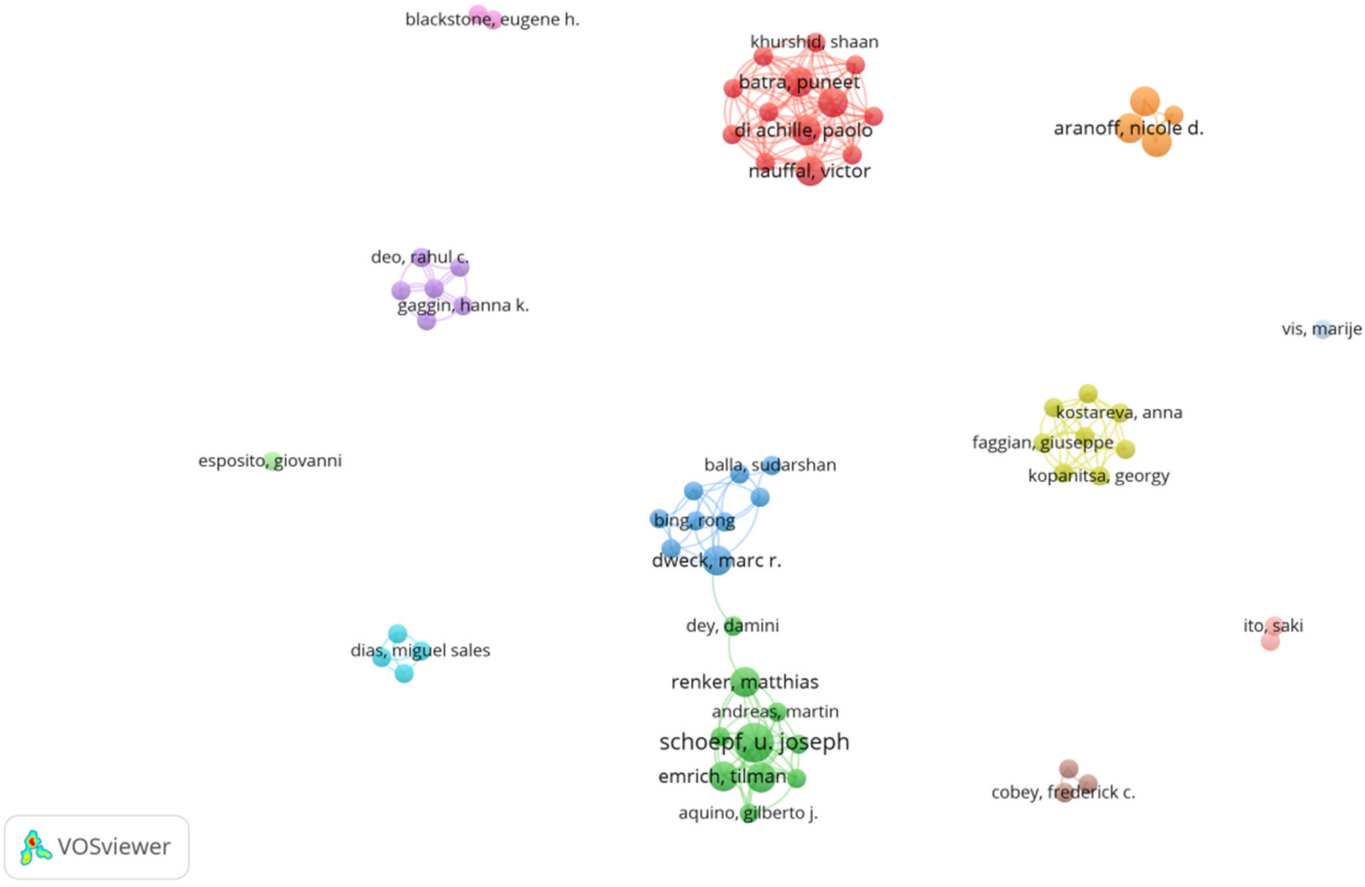
Cooperation map of authors in the studies of AI in aortic valve stenosis.

**Figure 4:**
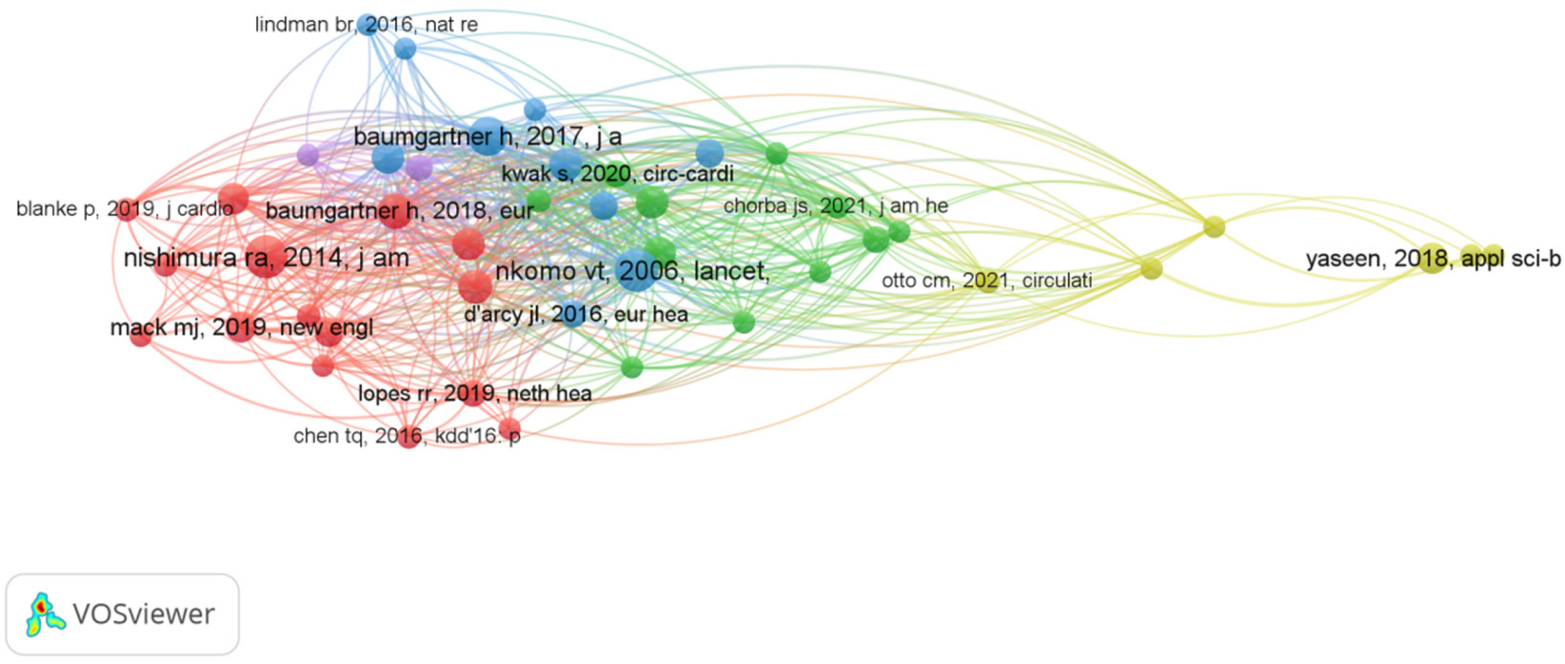
Co-reference network of AI in aortic valve stenosis.

Schoepf U. Joseph leads with four papers and 36 citations, primarily focusing on AI and coronary artery CT technologies (9) for optimizing preoperative assessment (10) and decision-making (11) for patients with severe AVS (12). The collaborative network among core authors indicates strong partnerships, particularly between Schoepf and Varga-Szemes, showing their pivotal role in advancing AI research in the field. From Figures 3, 4, a series of highly interconnected author clusters are evident, signifying the presence of stable research teams and cross-institutional collaborative projects.

#### 3.2.2 Analyzing major research institutions

Similar to the core author analysis, identifying principal research institutions focuses on assessing the institutions' publication output, citation frequency, and collaborative interactions. The University of California, San Francisco, Brigham & Women's Hospital, and Massachusetts General Hospital are the top research institutions, with the University of California leading in both the number of publications and citation impact in Table 3. These institutions are at the forefront of applying AI to improve the diagnosis and treatment of AVS, and their collaborations have significantly contributed to research advancements in this field.

**Table 3:** Top 9 organizations ranked by citations in AI applications in aortic valve stenosis research field.

Rank	Organization	Publications	Citations	ACI
1	University of California San Francisco	6	160	26.66
2	Mayo Clinic	4	121	30.25
3	Brigham & Women's Hospital	6	118	19.66
4	Northwestern University	4	110	27.5
5	Massachusetts General Hospital	5	102	20.4
6	Harvard Medical School	4	81	20.25
7	University of Edinburgh	4	75	18.75
8	Medical University of South Carolina	4	36	9
9	German Center for Cardiovascular Research	4	20	5

### 3.3 Geographical distribution and analysis of international cooperation

The United States dominates AI-AVS research with 47 papers and 595 citations, followed by Germany with 17 papers in Table 4, ranked by the number of citations. The distribution of publications across countries in this field is highly uneven, with a significant top-heavy effect.

**Table 4:** Top 5 countries ranked by citations in AI applications in aortic valve stenosis research field.

Rank	Country	Publications	Citations	ACI
1	USA	47	595	12.65
2	Canada	10	118	11.8
3	Germany	17	113	6.65
4	People's Republic of China	8	41	5.125
5	Italy	11	31	2.81

**Figure 5:**
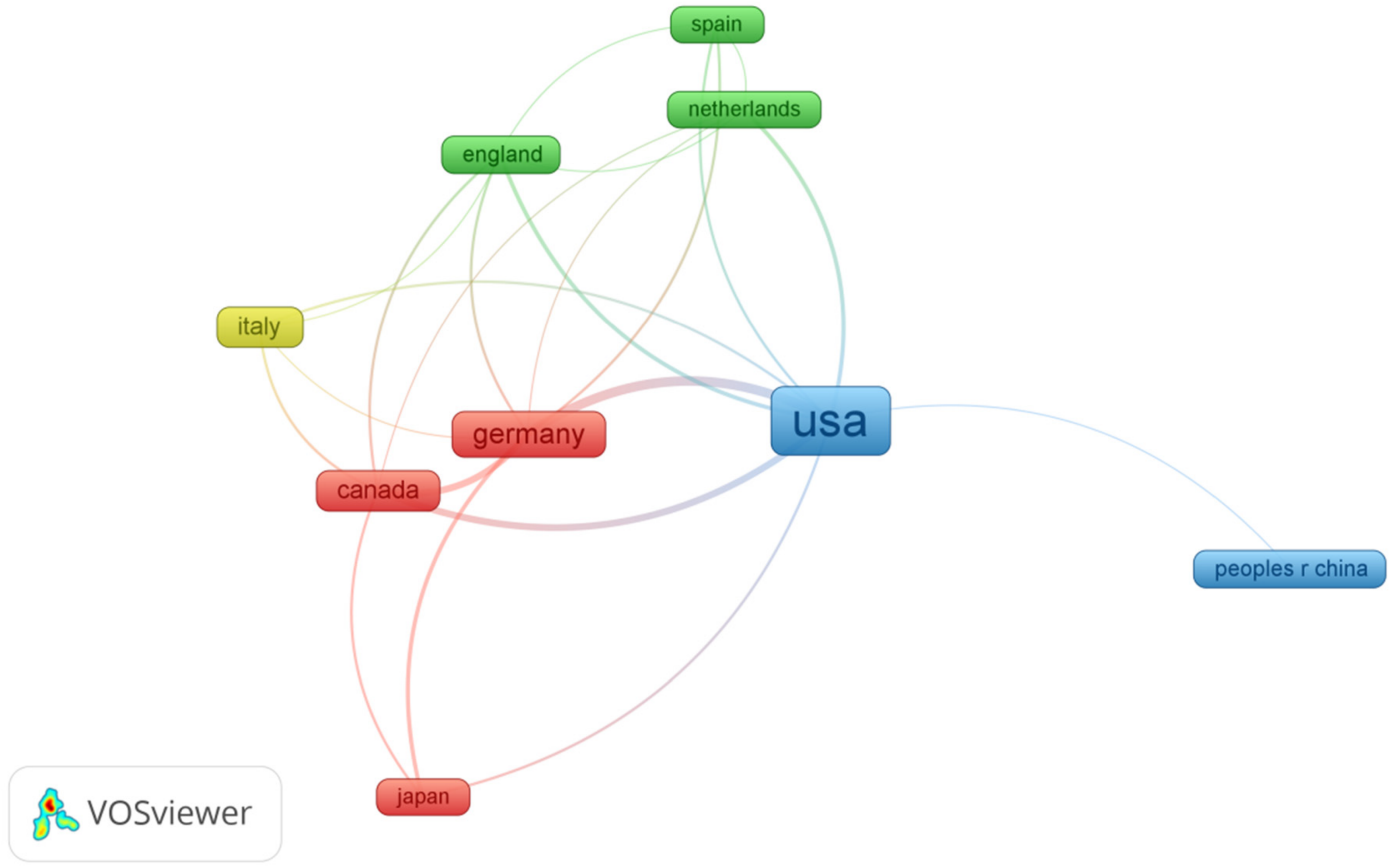
Cooperation map of countries in the studies of AI in aortic stenosis.

In Figure 5, the colours of the nodes in the international collaboration network analysis represent different clusters, with larger nodes indicating a higher volume of publications. International collaborations are particularly strong between the U.S., Germany, and Canada, fostering significant advancements. This cross-country collaboration has led to increased knowledge sharing and accelerated research progress in the AI-AVS domain.

### 3.4 Citation analysis: assessing the most influential articles, journals

#### 3.4.1 Analysis of highly cited literature

As shown in Table 5, the most cited article is “Deep Learning-Based Algorithm for Detecting Aortic Stenosis Using Electrocardiography,” where researchers demonstrated a deep learning-based algorithm capable of detecting severe Aortic Stenosis with high precision using 12-lead and single-lead electrocardiograms (ECG). The second most cited article developed an artificial intelligence-based electrocardiogram (AI-ECG) employing convolutional neural networks to identify patients with moderate to severe Aortic Stenosis. The results indicate that this AI-ECG exhibits high accuracy and has the potential to serve as a powerful screening tool for AVS in the community. These key studies demonstrate AI's transformative potential in AS diagnostics.

**Table 5:** Top 10 publications ranked by citations in AI applications in the aortic valve stenosis research field.

Rank	Author	Article Title	Journal	Year	Type	Citation
1	Kwon, J.M., et al. (13)	Deep learning-based algorithm for detecting aortic stenosis using electrocardiography	Journal of the American Heart Association	2020	Article	126
2	Yaseen, Y., G.Y. Son, et al. (14)	Classification of heart sound signal using multiple features	Applied Sciences-Basel	2019	Article	106
3	Cohen-Shelly, M., et al. (15)	Electrocardiogram screening for aortic valve stenosis using artificial intelligence	European Heart Journal	2021	Article	104
4	Goto, S., et al. (16)	Artificial intelligence-enabled fully automated detection of cardiac amyloidosis using electrocardiograms and echocardiograms	Nature Communications	2021	Article	102
5	Hernandez-Suarez, D.F., et al. (17)	Machine learning prediction models for in-hospital mortality after transcatheter aortic valve replacement	JACC: Cardiovascular Interventions	2019	Article	81
6	Gharehbaghi, A. and M. Linden (18)	A deep machine learning method for classifying cyclic time series of biological signals using time-growing neural network	IEEE Transactions on Neural Networks and Learning Systems	2018	Article	69
7	Casaclang-Verzosa, G., et al. (19)	Network tomography for understanding phenotypic presentations in aortic stenosis	JACC: Cardiovascular Imaging	2019	Article	60
8	Sengupta, P.P., et al. (20)	A machine-learning framework to identify distinct phenotypes of aortic stenosis severity	JACC: Cardiovascular Imaging	2021	Article	49
9	Kwak, S., et al. (21)	Unsupervised cluster analysis of patients with aortic stenosis reveals distinct population with different phenotypes and outcomes	Circulation-Cardiovascular Imaging	2020	Article	33
10	Lopes, R.R., et al. (22)	Value of machine learning in predicting TAVI outcomes	Netherlands Heart Journal	2019	Article	27

#### 3.4.2 Analyzing journal impact

The most influential journals in this field are Scientific Reports and Journal of the American Society of Echocardiography evaluated through metrics like the Journal Impact Factor (JIF) as shown in Table 6. These journals are central to disseminating research on AI applications in AVS, playing a vital role in the academic community's understanding of the potential of AI in clinical settings.

**Table 6:** Top 5 journals ranked by citations in AI applications in aortic valve stenosis research field.

Rank	Source	Publications	Citations
1	Scientific Reports	4	41
2	Journal of the American Society of Echocardiography	4	9
3	Frontiers in Cardiovascular Medicine	6	8
4	Diagnostics	5	4
5	Journal of Personalized Medicine	4	4

### 3.5 Analysis of research hot spots and frontier domains

#### 3.5.1 Keyword co-occurrence analysis identifies research hot spots

**Figure 6:**
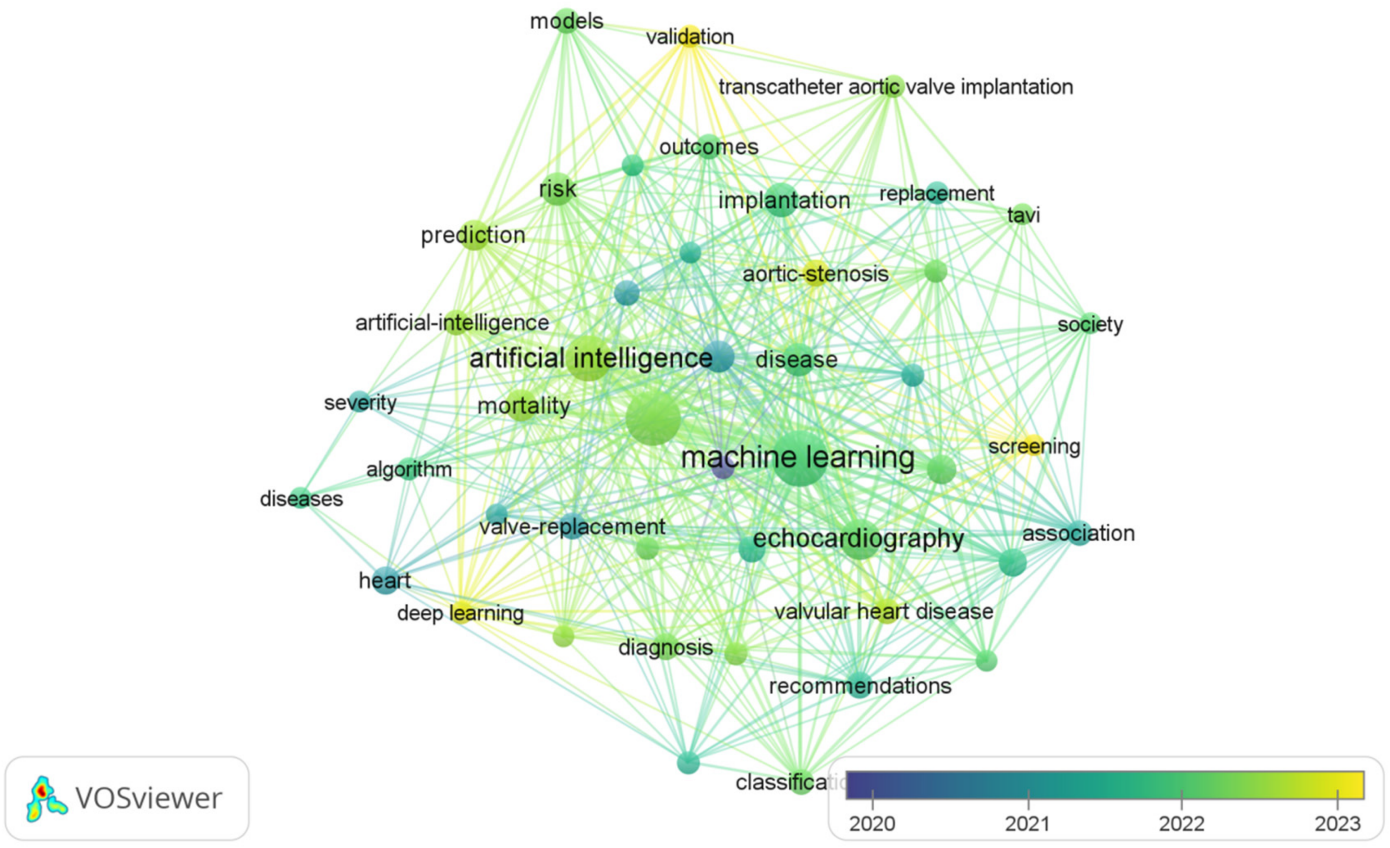
The overlay visualization of the co-occurrence of keywords.

**Figure 7:**
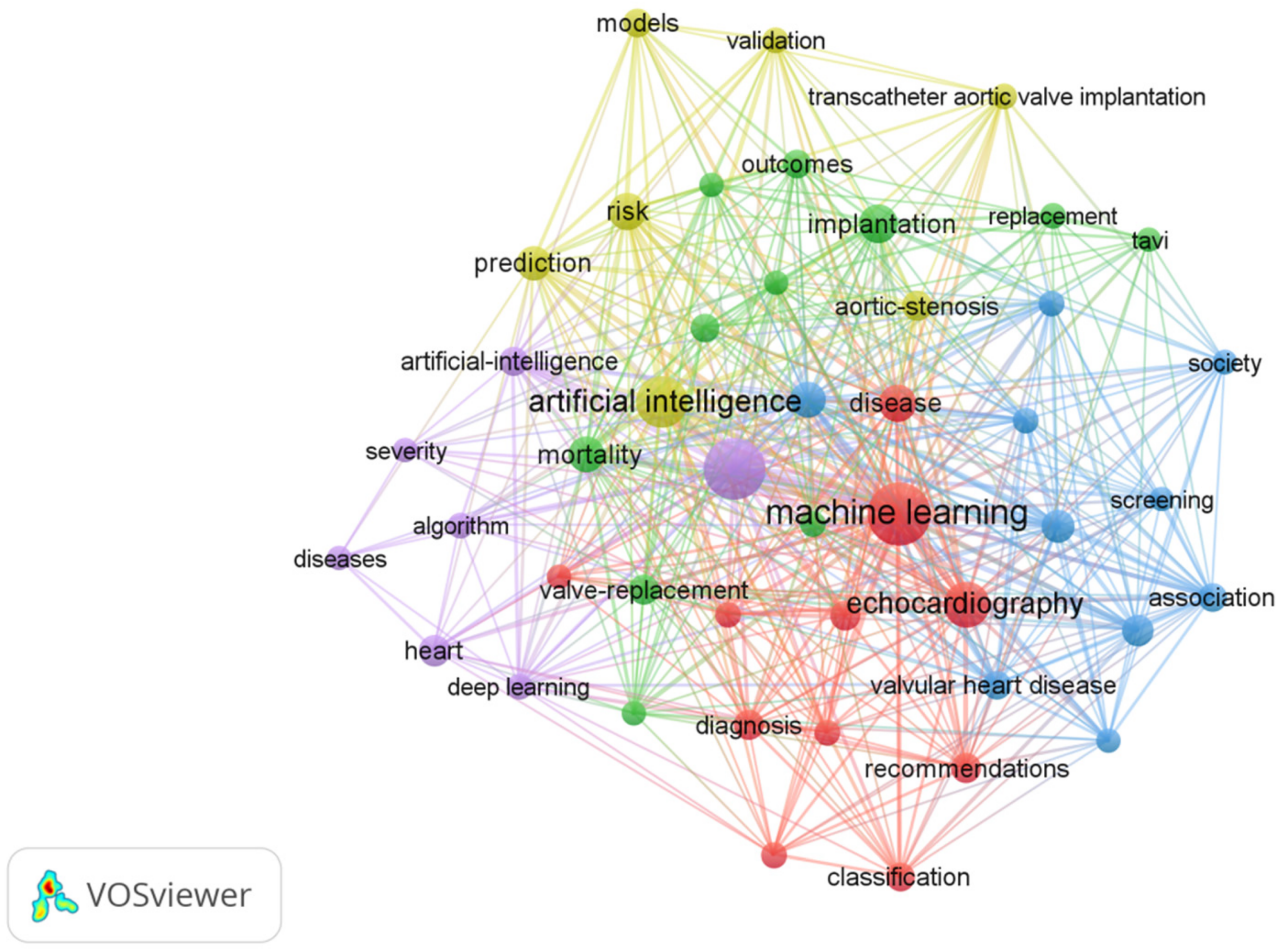
The network visualization of the co-occurrence of keywords.

The most frequently occurring keywords in the AI-AVS literature are “machine learning”, “aortic stenosis” and “artificial intelligence” in Table 7 signaling research focuses on diagnostic improvements and predictive models. Clusters of co-occurring keywords (Figures 6, 7) show that research is centered on AI applications in medical imaging, diagnosis, and risk prediction.

**Table 7:** Top 10 keywords in AI applications in aortic valve stenosis research field.

Rank	Keywords	Occurrences	Total link strength
1	machine learning	44	64
2	aortic stenosis	41	77
3	artificial intelligence	28	50
4	echocardiography	22	37
5	implantation	15	15
6	risk	13	30
7	disease	13	25
8	mortality	12	28
9	stenosis	12	23
10	prediction	11	23

As illustrated in Table 8, keywords such as “machine learning,” “implantation,” “management,” “artificial intelligence,” and “aortic stenosis” stand out due to their high frequency. These discoveries offer a lucid perspective on the research trends and core themes prevalent in the field.

**Table 8:** Cluster of keywords in AI applications in aortic valve stenosis research field.

Cluster	Colour	Label	Keywords
1	Red	Machine learning in cardiac imaging and disease classification	Machine learning, calcification, classification, diagnosis, disease, dysfunction, echocardiography, heart failure, impact, quantification, recommendations
2	Green	TAVI and imaging-based clinical outcomes	Implantation, computed-tomography, ejection fraction, mortality, outcomes, replacement, segmentation, tavi, transcatheter aortic valve replacement, valve-replacement
3	Blue	Management and screening of aortic stenosis	Management, accuracy, aortic-valve-replacement, association, prevalence, screening, society, stenosis, valvular heart disease
4	Yellow	AI for risk prediction and personalized treatment	Artificial intelligence, aortic stenosis, models, prediction, risk, transcatheter aortic valve implantation, validation
5	Purple	Deep learning for disease severity assessment and prognosis	Aortic stenosis, algorithm, artificial intelligence, deep learning, diseases, heart, severity

#### 3.5.2 Red cluster (Part 1)

AI in cardiac imaging and disease classification, emphasizing machine learning techniques to enhance echocardiographic analysis and improve disease classification accuracy. “Machine learning” as a potent tool for data analysis, is being widely applied in the classification, diagnosis, quantitative analysis, and quantification of the impact of heart diseases. We observed that “machine learning” shares a cluster with keywords such as “calcification”, “classification”, “diagnosis”, “disease”, “dysfunction”, “echocardiography”, “heart failure”, “impact”, “quantification” and “recommendations” in the keyword co-occurrence network. This clustering indicates that the application of machine learning technology in the fields of heart calcification, disease classification, and diagnosis has garnered widespread attention (23, 24). Moreover, the co-occurrence of terms like “echocardiography”, “heart failure” and “dysfunction” further underscores the significance of machine learning in evaluating cardiac function and researching heart failure (25). For instance, in the detection and quantification of cardiac calcification, machine learning is capable of processing complex imaging data to reveal pathological features and their association with aortic stenosis. Additionally, “echocardiography” an essential diagnostic tool in cardiology, benefits from the application of machine learning, enhancing diagnostic accuracy, particularly in evaluating cardiac dysfunction and heart failure. Studies have shown that the data-driven insights provided by machine learning have a significant impact on forming treatment recommendations and clinical practice guidelines (19). In summary, “machine learning” serves as a nexus connecting several key research areas in cardiology, heralding a new era in cardiac research and practice. Through in-depth analysis of this cluster, researchers can better understand how machine learning optimizes the diagnostic and treatment pathways for heart diseases and how it leads the future trends in medical research.

#### 3.5.3 Green cluster (Part 2)

TAVI and imaging-based clinical outcomes, focusing on the role of imaging technologies like computed tomography in evaluating outcomes of transcatheter valve implantation. Analysis of the keyword co-occurrence network reveals a distinct cluster closely associating “Implantation” with a series of related technologies and clinical outcomes in the domain of aortic stenosis, indicating that utilizing computed tomography (CT) for cardiac structural imaging segmentation, assessing ejection fraction, and predicting mortality and other clinical outcomes are current hotspots in research and clinical practice, especially when employing transcatheter aortic valve implantation (TAVI) strategies (9). The formation of this cluster not only emphasizes the central role of imaging in the pre and post-assessment of AVS treatment (26) but also highlights the importance of multidisciplinary approaches in improving patient prognosis (22). Thus, focusing on the literature within this domain is crucial for enhancing the success rate of AVS treatments and improving long-term patient outcomes. Further analysis shows that within this cluster, not only do the keywords co-occur frequently, but their mutual citations in the literature also prove their technological and conceptual interconnections and dependencies, together constituting a comprehensive and multidimensional research domain in AVS studies.

#### 3.5.4 Blue cluster (Part 3)

Management and screening of aortic stenosis, addressing guidelines, prevalence, and early detection strategies.The keyword co-occurrence analysis of literature on aortic stenosis management strategies has revealed a tight network of associations between the word “Management” and other keywords such as “accuracy”, “aortic-valve-replacement”, “association”, “prevalence”, “screening”, “society”, “stenosis” and “valvular heart disease”. This cluster reflects the comprehensive management needs of AVS patients in medical practice, including ensuring the precision of treatments and interventions (27), understanding the prevalence of the condition (28), enhancing early screening (29), and assessing the outcomes of cardiac valve replacement surgeries (3). Therefore, the research suggests that utilizing the keywords within this cluster can promote a multifaceted management strategy for AVS, which is crucial for improving clinical outcomes.

#### 3.5.5 Yellow cluster (Part 4)

AI for risk prediction and personalized treatment, highlighting the use of predictive modeling to assess risks and validate AI frameworks in AVS management. In the modern treatment and research of aortic stenosis, artificial intelligence, as an innovative technology, is forming a tight interactive network with traditional clinical keywords such as “accuracy”, “screening” and “disease prevalence trends.” The growing application of artificial intelligence in medical image recognition, pathological prediction, and patient management, is demonstrating immense potential in the diagnosis and treatment of cardiac valve diseases, especially aortic stenosis. Moreover, the integration of artificial intelligence technology not only enhances the accuracy and efficiency of disease management (20) but also promotes data-driven decision-making in developing screening guidelines and treatment recommendations by public health organizations and professional associations. Consequently, artificial intelligence plays an increasingly important role in optimizing disease detection (30), therapeutic interventions (31, 32), and improving patient quality of life (33), signalling a significant transformation in the research and treatment methodologies of cardiac valve diseases and opening a new chapter in personalized medicine and precision treatment (34).

#### 3.5.6 Purple cluster (Part 5)

Deep learning for disease severity assessment and prognosis, exploring how deep learning is applied to evaluate cardiac function and AS severity. The application of artificial intelligence and deep learning technologies in the assessment of the severity of aortic stenosis pathology and treatment decisions is increasingly prevalent. This cluster reveals how researchers employ complex algorithms to deepen their understanding of aortic stenosis and other cardiac diseases (13), and develop tools capable of accurately assessing disease severity and predicting patient prognosis (35). This not only demonstrates the potential of deep learning in disease classification and prognosis prediction but also highlights the significant role of artificial intelligence in the diagnosis and treatment of cardiovascular diseases, especially against the backdrop of the rapid development of non-invasive diagnostic technologies (36–38). These interdisciplinary technological advancements offer new perspectives for personalized medicine in cardiac diseases and could drive the management of cardiac diseases towards more precise and efficient directions.

### 3.6 Integrated evolutionary path of the literature

**Figure 8:**
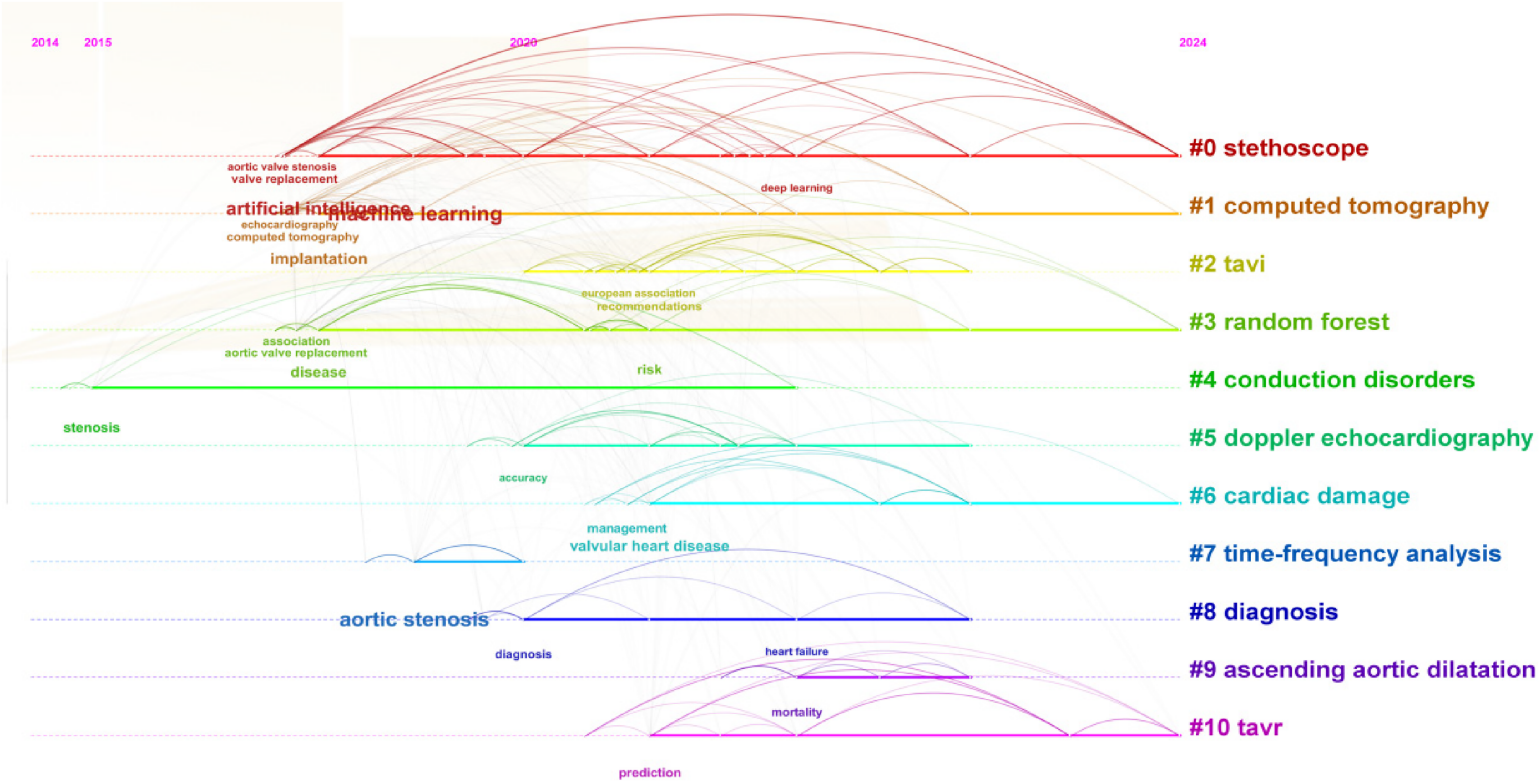
Evolutionary path in the studies of AI in aortic valve stenosis.

The evolution of AI research in AVS shows a shift from traditional diagnostic tools, such as stethoscopes, to advanced AI-driven methods like deep learning and machine learning models in Figure 8. This transition particularly underscores the advancements in cardiac disease diagnosis and treatment, from traditional imaging techniques (such as computed tomography and echocardiography) to state-of-the-art interventional procedures (like TAVI and TAVR), which have significantly enhanced diagnostic and therapeutic efficiencies.These AI technologies have greatly enhanced the accuracy of AVS diagnostics and treatment, indicating a trend toward more personalized care.

### 3.7 Analysis of academic growth points

**Figure 9:**
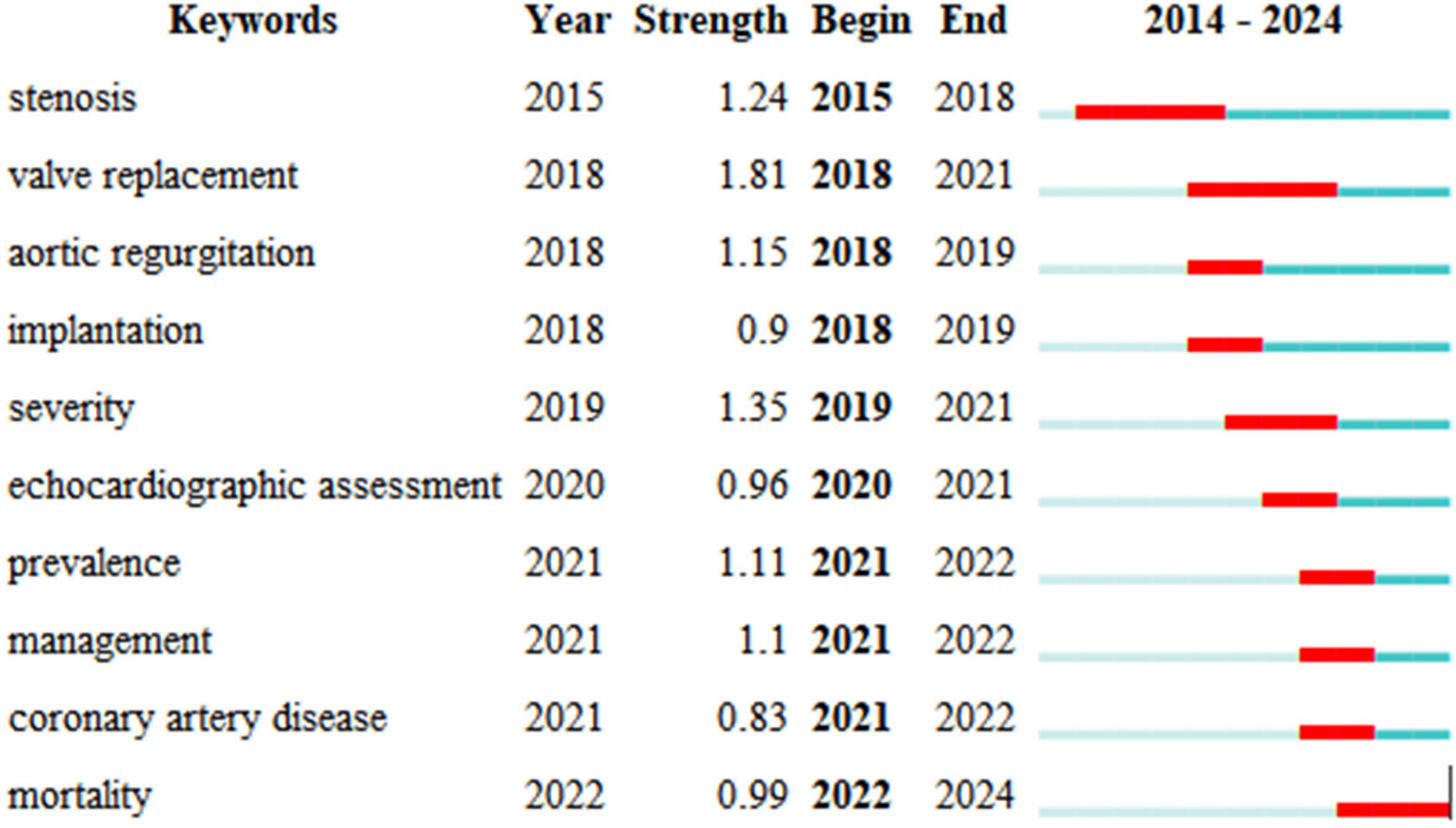
Top 10 keywords with the strongest citation bursts.

Keyword citation bursts highlight the growing importance of topics like “valve replacement” and “stenosis,” reflecting increasing attention on AI's role in improving diagnostic accuracy and treatment outcomes. As illustrated in Figure 9, these pivotal terms not only mirror the shifting paradigms of research trends but also signify the scientific community's escalating engagement with certain queries, offering profound insights into the research focal points and the academic shift of attention within this sphere. Emerging themes such as “management” and “coronary artery disease” suggest future research will focus on optimizing patient care and reducing the burden of cardiovascular diseases.

## 4 Discussion

Through bibliometric analysis, our research highlights the significant advances in applying AI technology to the diagnosis and treatment of AVS. AI has shown great promise in improving diagnostic accuracy through automated image analysis and predictive modeling. These tools allow clinicians to process complex imaging data efficiently, extracting crucial information that aids in early diagnosis and treatment planning (39). AI applications in echocardiography and ECG have shown promise in research for reducing errors and personalizing treatment options. However, these applications have not yet been widely integrated into clinical practice.

Our findings align with previous bibliometric analyses that have explored aortic valve disease research. For example, Wang et al. (8) highlighted anticoagulation management and frailty assessment as critical areas for improving outcomes after valve replacement, while Fang et al. (7) identified TAVI as a central focus in research trends, emphasizing its expanding implementation and clinical challenges. Guzel et al. (40) provided insights into the most cited studies on TAVI, shedding light on influential research and its geographical distribution. Additionally, Wang et al. (41) analyzed inflammatory mechanisms in aortic disease, identifying calcification and oxidative stress as major research hotspots. Our analysis uniquely emphasizes the role of artificial intelligence in AVS research. By identifying key contributors, institutions, and emerging themes such as machine learning in cardiac imaging and AI-based predictive modeling, this study bridges an important gap in the literature.

Despite these advancements, several challenges remain. AI models, particularly those based on deep learning, often lack transparency, making them difficult to interpret in clinical settings. This “black box” issue presents a barrier to the widespread adoption of AI, as clinicians need to understand how decisions are made to fully trust these technologies. Additionally, data privacy concerns arise as AI systems often require large datasets containing sensitive patient information. AI's implementation in real-life clinical practice remains limited. This gap underscores the importance of transitioning AI models from experimental research settings to clinical applications. Overcoming barriers such as regulatory approval, data security, and model validation in real-world scenarios will be essential for broader adoption. Addressing these challenges represents a critical focus for future research and development efforts.

While data from 2024 are included in this analysis, they represent only the first quarter of the year. As such, findings related to 2024 should be interpreted cautiously, as they may not fully capture publication trends for the entire year. Moreover, the research hotspots identified—such as machine learning in cardiac imaging and AI-based predictive models—point to a rapidly evolving field. These areas reflect the growing interest in developing non-invasive, AI-driven tools to enhance the accuracy of AVS diagnostics and treatment outcomes (20, 42). As AI technology continues to advance, it is likely to become an integral part of cardiovascular care, enabling more precise and personalized treatment strategies. Future research should focus on improving AI model interpretability, ensuring data security, and expanding AI's application across different clinical scenarios. Addressing these challenges will help to further integrate AI into the clinical workflow, ultimately improving patient outcomes and reducing the burden of cardiovascular diseases.

## 5 Conclusions

AI is increasingly applied to the diagnosis and treatment of AVS, particularly in areas like cardiac imaging and risk assessment. Machine learning and deep learning technologies have significantly enhanced diagnostic precision and personalized treatment approaches. However, challenges such as model transparency and data security still require attention. Future research should prioritize improving AI model interpretability and ensuring safe data usage, while fostering international collaboration to drive further advancements in this field.

## Data Availability

The original contributions presented in the study are included in the article/Supplementary Material, further inquiries can be directed to the corresponding author/s.
